# Evaluating the field performance of multiple SARS-Cov-2 antigen rapid tests using nasopharyngeal swab samples

**DOI:** 10.1371/journal.pone.0262399

**Published:** 2022-02-14

**Authors:** Magyar Nóra, Dániel Déri, Dániel Sándor Veres, Zoltán Kis, Erzsébet Barcsay, Bernadett Pályi

**Affiliations:** 1 National Biosafety Laboratory, National Public Health Center, Budapest, Hungary; 2 Schools of Doctoral Studies, Semmelweis University, Budapest, Hungary; 3 Department of Virology, National Public Health Center, Budapest, Hungary; 4 Doctoral School of Biology, Institute of Biology, ELTE Eötvös Loránd University, Budapest, Hungary; 5 Department of Biophysics and Radiation Biology, Semmelweis University, Budapest, Hungary; 6 Department of Microbiology, Semmelweis University, Budapest, Hungary; Qatar University, QATAR

## Abstract

The SARS-CoV-2 pandemic, which started in December 2019, has been posing significant challenges to the health care system worldwide. As the pandemic spreads with rapidly increasing number of positive cases, early diagnosis of infected patients is crucial to successfully limit the spread of the virus. Although the real-time reverse-transcription polymerase chain reaction (RT-qPCR) is the recommended laboratory method to diagnose COVID-19 infection, many factors such as availability of laboratory equipment, reagents and trained personnel affect the use of time-consuming molecular techniques. To facilitate on-the-spot diagnosis of COVID-19, SARS-CoV-2 rapid antigen tests were developed by several different manufacturers. The evaluation of such rapid tests is particularly important due to the recent unanimous agreement by the European Commission Member States on a recommendation setting out a framework for the use of antigen rapid tests that contains a list of the mutually recognized assays and the basis of independent validation protocols. To evaluate the on-field performance of ten commercially available SARS-CoV-2 antigen rapid tests (CLINITEST Rapid COVID-19 Antigen Test, GenBody COVID-19 Antigen Test, GENEDIA W COVID-19 Ag Test, Healgen Coronavirus Antigen Rapid Test, Humasis COVID-19 Ag Test, VivaDiag SARS-CoV-2 Ag Rapid Test, Helix i-SARS-CoV-2 Ag Rapid Test, Roche SARS-CoV-2 Rapid Antigen Test, Abbot COVID-19 Ag Rapid Test and Vazyme SARS-CoV-2 Antigen Detection Kit) and compare with RT-qPCR as a reference method, the Hungarian National Public Health Center provided 1,597 antigen rapid tests to the National Ambulance Service, COVID-testing trucks and two hospitals treating COVID-19 patients. Sensitivity, specificity and accuracy were determined by performing the rapid test directly from nasopharyngeal swab samples of symptomatic individuals. For strongly positive samples (Ct < 25) sensitivities ranged between 66.7% and 100%, while for positive samples (Ct < 30) they gave a maximum sensitivity of 87.5%. The specificity of the tests was ranging between 79% to 100%. The results presented here are of high importance to the European Commission and also help governmental decision-making regarding the application of the proper rapid tests for screening different at-risk populations. Nonetheless, SARS-Cov-2 rapid tests play an important role in early and on-the-spot diagnosis of potentially infected individuals.

## Introduction

At the end of 2019, a novel respiratory disease with unknown etiology appeared in Wuhan, China. A few weeks later in January 2020, China announced that it is caused by a novel coronavirus named severe acute respiratory syndrome coronavirus 2 (SARS-CoV-2) [1]. Due to the rapid spatiotemporal spreading, it was declared as a pandemic by the World Health Organization (WHO) in March 2020.

In Hungary, the first diagnosed cases occurred also in March 2020, and by this date over 800,000 people got infected (in a country with population of 9.7 million) [2]. From the beginning, over 5.8 million quantitative reverse transcription polymerase chain reaction (RT-qPCR) tests were performed in national and private laboratories countrywide until early June 2021 [3].

Although RT-qPCR is the recommended laboratory method with the highest sensitivity and specificity to diagnose COVID-19 infection, molecular tests require availability of proper laboratory equipment, reagents, trained personnel and strictly regulated shipment of clinical samples [4–6]. Furthermore, to prevent the fourth pandemic wave, we need to increase the number of tests even more than before–besides the indisputably important vaccination campaign. To maintain and facilitate an adequate testing capacity, on-field diagnostic methods e.g., antigen-based rapid test devices (Ag-RTD) are indispensable tools beside the gold standard RT-qPCR. In fact, Ag-RTDs will never substitute the RT-qPCR in sensitivity but using them as a tool for point-of-care testing proved to be remarkably helpful for hospitals, ambulance services and mass testing. As Ag-RTDs are easy to perform and utilizing them allows to process a large number of different types of samples within a relatively short time, with the adequate sensitivity they are ideal for testing symptomatic patients and for on-field screening (e.g., in schools or university settings, also employees in offices or factories). According to the interim guidance of the Centers for Disease Control and Prevention (CDC), this is a well-founded strategy, as screening makes asymptomatic infections easier to identify and interrupts viral transmission routes, especially in the case of high community risks. Negative Ag-RTD results should be strengthened by RT-qPCR, especially in the case of symptomatic cases; however, SARS-CoV-2 positivity within the recommended time interval means proven infection among asymptomatic carriers as well, considering false positivity is likely impossible using a properly inspected and certified (In Vitro Diagnostic–IVD) device [7].

The aim of our study was to evaluate the on-field performance of ten different, commercially available SARS-CoV-2 antigen-based rapid test devices optimized for nasopharyngeal samples among symptomatic patients and compare them to the recommended method RT-qPCR.

## Materials and methods

### Study setup and clinical samples

Between 1 October 2020 and 28 February 2021, the National Public Health Center (NPHC) provided ten different SARS-CoV-2 antigen rapid tests to the National Ambulance Service, COVID testing trucks (operated by NPHC) and two hospitals treating COVID-19 patients (Országos Korányi Pulmonológiai Intézet Budapest and Szent János Kórház és Észak-budai Egyesített Kórházak, Budapest) (ethical approval number OGYÉI/58141/2020). During the strictly regulated routine sampling procedure, nasopharyngeal swab samples were collected in parallel for on-the-spot antigen rapid testing from the left nostril and left part of the throat and taken into test-specific sample buffers. For viral nucleic acid amplification (NAAT) tests samples were taken into viral transport medium (VTM) from the right part of the same respiratory area by health care professionals. A total number of 1,572 rapid testing and RT-qPCR test sampling was performed in parallel. All volunteers were informed and signed a consent form to participate. Results of the antigen tests (readout within the recommended time intervals) and the clinical specimens in VTM were transferred to the NPHC COVID-19 laboratory where the RT-qPCR was performed. Results were evaluated at the NPHC.

### Antigen rapid testing

In this study, ten different commercially available SARS-CoV-2 antigen rapid tests were included (Table 1).

**Table 1:** List of tested SARS-CoV-2 Ag-RTDs and manufacturers.

No	Name	JRC* ID [19]	CE marking	Detected antigen	Distributor
1	CLINITEST Rapid COVID-19 Antigen Test	1218	yes	nucleocapsid	Siemens Healthineers
2	GenBody COVID-19 Ag Test	1244	yes	n.a.	GenBody, Inc
3	GENEDIA W COVID-19 Ag Test	1144	yes	nucleocapsid	Green Cross Medical Science Corp.
4	Humasis COVID-19 Ag Test	1263	yes	nucleocapsid + RBD	Humasis
5	VivaDiag SARS-CoV-2 Ag Rapid Test	1246	yes	nucleocapsid	VivaChek Biotech
6	Helix i-SARS-CoV-2 Ag Rapid Test	na	yes	nucleocapsid	Cellex Biotech Co.
7	SARS-CoV-2 Antigen Rapid Test SARS-CoV-2 Rapid Antigen Test	1604	yes	nucleocapsid	Roche
8	Rapid COVID-19 Antigen Test	1735	yes	nucleocapsid	Healgen Scientific
9	Panbio COVID-19 Ag Rapid Test	1232	yes	nucleocapsid	Abbott Rapid Diagnostics
10	Severe Acute Respiratory Syndrome Coronavirus 2 (SARS-CoV-2) Antigen Detection Kit (Colloidal Gold-Based)	1849	yes	nucleocapsid	Nanjing Vazyme Medical Technology Co

All tests allow qualitative detection of SARS-CoV-2 virus antigens in human nasopharyngeal swab samples. Rapid testing was performed on field according to manufacturer’s instructions by trained health care personnel. Results were evaluated after the recommended incubation time and sent to the NPHC COVID-19 laboratory.

*European Commission Joint Research Center (JRC).

### Nucleic acid extraction and RT-qPCR

Nucleic acid extraction and RT-qPCR were performed in the NPHC COVID-19 laboratory. Viral RNA was isolated from 200 μL of the clinical specimens using the Roche MagNA Pure 96 Instrument (Roche Life Sciences, Basel). To detect viral nucleic acid RT-qPCR was conducted on the LightCycler 480 Instrument II platform using the PerkinElmer® SARS-CoV-2 Real-time RT-PCR Assay (PerkinElmer Inc., Waltham, Massachusetts, United States) to detect SARS-CoV-2 ORF1ab and N genes and human Rnase P gene as internal control (IC). RT-qPCR was performed according to the manufacturer’s instructions. Samples positive for both SARS-CoV-2 specific genes (with Ct values < 40.0) and the internal control were considered positive. Samples that gave negative results for both ORF1ab and N genes but positive for human IC qualified as negative. RT-qPCR was concluded as inconclusive if positive or inconclusive only for one SARS-CoV-2 gene.

### Statistical analysis

Statistical analysis was performed using R statistical software [8]. Overall sensitivity, specificity, accuracy, positive and negative predictive values of Ag-RTDs were estimated with 95% exact binomial confidence interval (upper limit is truncated to 1, if >1) using EpiR package (version 2.0.19). Sensitivity was also calculated in the case of RT-qPCR positive samples with Ct values ≤ 25 and ≤ 30 with 95% confidence interval.

The effect of Ct value on Ag-RTD result was estimated by a logistic regression model (using logit link). The final model was the following. The antigen test result as the outcome variable was explained by the Ct value. The effect was adjusted for the type of antigen test, age, and sex. There was no interaction term in the final model. The model fit was checked by simulation model diagnostic plots using DHARMa package (version 0.3.3.0).

## Results

### Sample collection and testing the study groups

An overall number of 1,572 rapid testing with 10 different Ag-RTDs and RT-qPCR sampling was performed parallel, by the National Ambulance Service, COVID testing trucks (operated by NPHC) and two COVID-19 hospitals in Budapest. The outcome of the Ag-RTD was unclear in the case of 4 cases, and RT-qPCR gave inconclusive result with 13 samples. Those samplings were discarded from the statistical analysis resulting a total number of 1,557 enrolled cases. Mean age in the study group was 41.0 years (Table 2). An overall number of 858 (55.1%) female and 699 (44.9%) male individuals were enrolled. Three hundred and forty (21.8%) samples were Ag-RTD positive, and 1,217 (78.2%) resulted as negative. When testing the samples with RT-qPCR, 570 (36.6%) showed nucleic acid positivity, and 987 (63.4%) were negative for SARS-CoV-2. When comparing the study groups in the case of all 10 rapid tests, the average age of the tested individuals was between 35.2 and 43.0 years, and the ratio of male individuals was between 42.3–49.4%.

**Table 2:** Age and gender distriubion in the study group and the number of Ag-RTD and RT-qPCR positive and negative samples.

	Abbott (N = 543)	Clinitest (N = 99)	Genbody (N = 98)	Genedia (N = 97)	Healgen (N = 87)	Helix (N = 96)	Humasis (N = 97)	Roche (N = 199)	Vazyme (N = 120)	Vivadiag (N = 121)	Overall (N = 1557)
**Age**											
Mean (SD, CV%)	43.0 (18.8, 43.7%)	41.8 (15.8, 37.8%)	40.4 (17.0, 42.1%)	43.0 (16.8, 39.1%)	41.7 (15.6, 37.5%)	35.2 (16.3, 46.3%)	40.2 (15.6, 38.8%)	39.8 (18.4, 46.2%)	40.3 (16.4, 40.7%)	37.9 (16.8, 44.3%)	41.0 (17.6, 42.9%)
Median (IQR)	43.1 (26.4)	42.2 (19.4)	42.8 (24.3)	46.8 (23.7)	43.7 (19.5)	37.0 (22.6)	42.7 (22.2)	42.2 (26.5)	42.4 (20.6)	39.3 (21.0)	42.3 (24.4)
Min, Max	4.49, 93.1	1.20, 87.9	3.67, 86.8	4.10, 72.8	2.43, 73.1	4.68, 70.7	0.00, 73.7	4.88, 87.4	4.11, 85.9	4.65, 82.8	0.00, 93.1
**Sex**											
female	311 (57.3%)	51 (51.5%)	51 (52.0%)	56 (57.7%)	44 (50.6%)	51 (53.1%)	54 (55.7%)	112 (56.3%)	65 (54.2%)	63 (52.1%)	858 (55.1%)
male	232 (42.7%)	48 (48.5%)	47 (48.0%)	41 (42.3%)	43 (49.4%)	45 (46.9%)	43 (44.3%)	87 (43.7%)	55 (45.8%)	58 (47.9%)	699 (44.9%)
**Ag-RTD result**											
positive	91 (16.8%)	24 (24.2%)	31 (31.6%)	22 (22.7%)	19 (21.8%)	24 (25.0%)	33 (34.0%)	47 (23.6%)	34 (28.3%)	15 (12.4%)	340 (21.8%)
negative	452 (83.2%)	75 (75.8%)	67 (68.4%)	75 (77.3%)	68 (78.2%)	72 (75.0%)	64 (66.0%)	152 (76.4%)	86 (71.7%)	106 (87.6%)	1217 (78.2%)
**PCR result**											
positive	176 (32.4%)	48 (48.5%)	40 (40.8%)	38 (39.2%)	33 (37.9%)	44 (45.8%)	36 (37.1%)	73 (36.7%)	47 (39.2%)	35 (28.9%)	570 (36.6%)
negative	367 (67.6%)	51 (51.5%)	58 (59.2%)	59 (60.8%)	54 (62.1%)	52 (54.2%)	61 (62.9%)	126 (63.3%)	73 (60.8%)	86 (71.1%)	987 (63.4%)
**Ct**											
Mean (SD, CV%)	28.3 (6.30, 22.2%)	25.9 (6.64, 25.6%)	24.4 (5.96, 24.4%)	25.2 (6.07, 24.1%)	27.3 (6.45, 23.6%)	27.2 (5.50, 20.2%)	27.6 (6.22, 22.5%)	27.2 (5.86, 21.5%)	25.6 (4.30, 16.8%)	28.0 (6.46, 23.1%)	27.1 (6.13, 22.7%)
Median (IQR)	28.8 (11.4)	24.8 (12.8)	24.2 (11.1)	24.5 (11.0)	27.1 (10.7)	26.5 (7.79)	28.2 (8.41)	26.2 (9.83)	25.5 (6.15)	28.1 (12.4)	26.8 (10.6)
Min, Max	13.0, 37.9	13.2, 36.6	14.0, 34.5	16.2, 36.2	14.2, 37.3	16.7, 38.1	13.5, 36.6	18.0, 37.4	18.4, 35.0	17.9, 36.8	13.0, 38.1

### Performance of the Ag-RTDs

**Fig 1:**
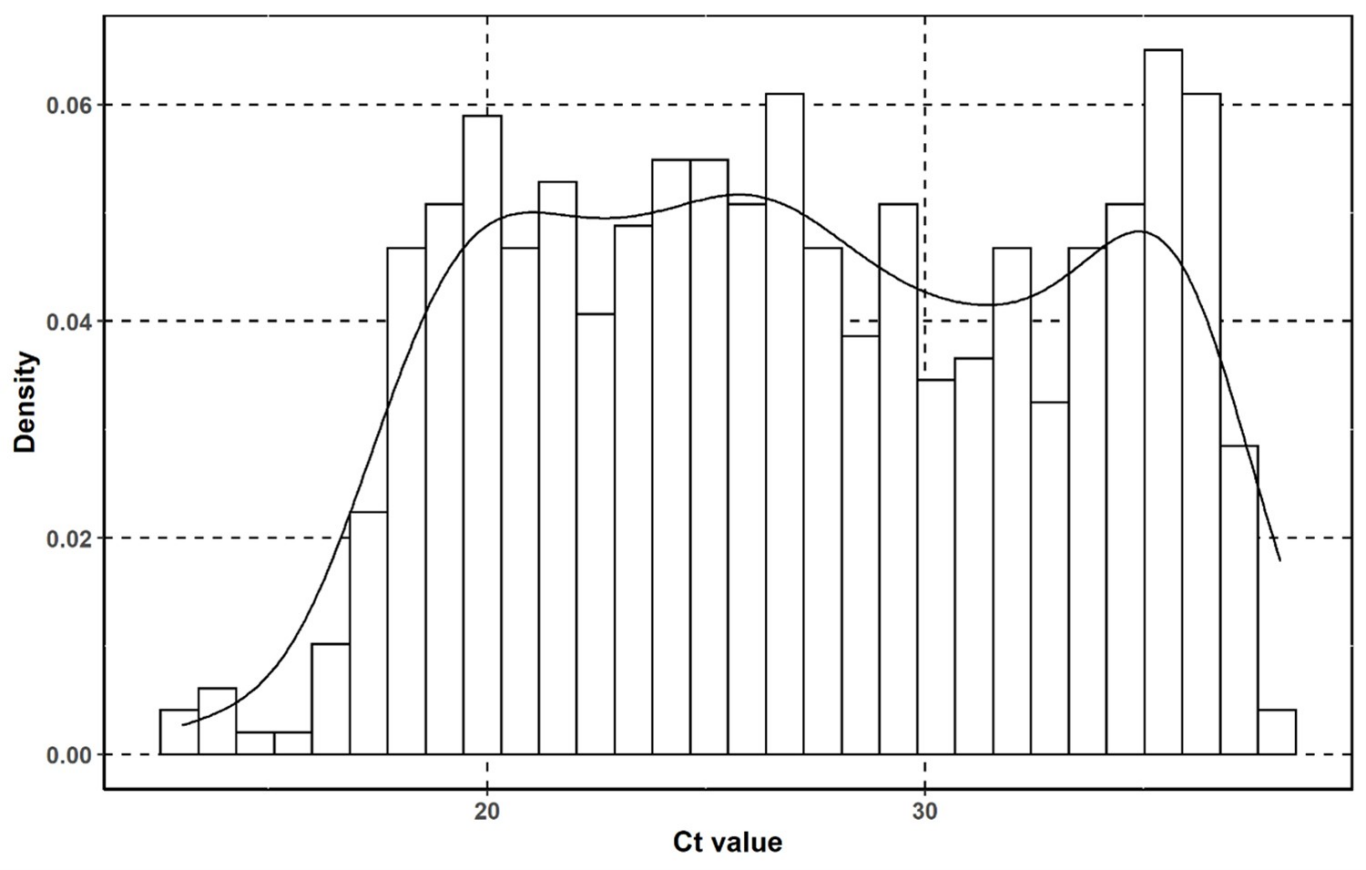
Ct value distribution of the RT-qPCR samples in the whole study group on a histogram with kernel density curve.

To be able to compare the different Ag-RTDs, it is important to first determine the Ct value distributions measured by RT-qPCR, as Ct value is a semi-quantitative value to calculate viral load in the samples. The mean Ct value (Table 2) was 27.1 (SD 6.13, CV% 22.7) with the minimum (highest viral load) of 13.0 and the maximum (lowest viral load) of 38.1 (Fig 1).

When comparing the RT-qPCR positive samples in the case of the different Ag-RTDs, the mean Ct values were between 24.4 (SD 5.96, CV% 25.6%) and 28.3 (SD 6.30, CV% 22.3). The ratio of true positive (RT-qPCR positive) samples was between 28.9–48.5% (average 36.6%).

**Fig 2:**
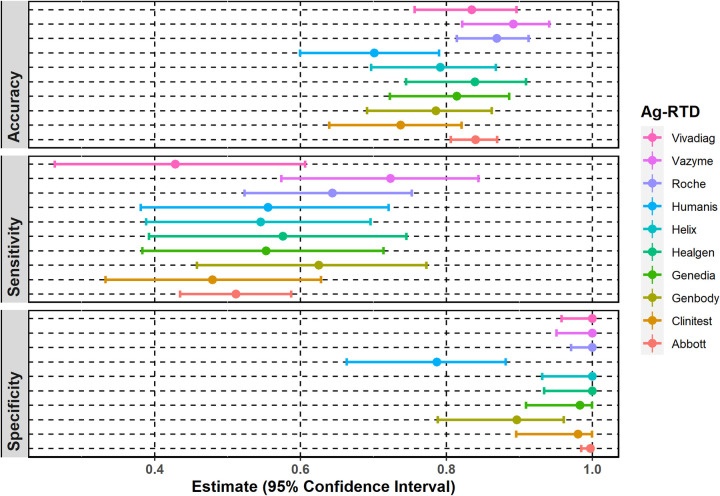
Sensitivity, specificity, and accuracy of each examined antigen rapid test. Point estimates with 95% exact binomial confidence intervals for sensitivity and specificity.

To evaluate on-field performance, overall sensitivity, specificity, and accuracy were calculated in the case of each Ag-RTD with 95% confidence intervals (CI 95%). However, the number of samples tested with the Ag-RTDs was different (N = 87–543), as we had more Abbot COVID-19 Ag Rapid Tests available to evaluate (Table 2). Sensitivity and specificity data are shown in Fig 2.

Overall sensitivity and specificity of the Ag-RTDs is 56.0% (CI 95%: 52.0–60.0%) and 98.0% (CI 95%: 97.0–99.0%), respectively. The highest sensitivity (72.0%, CI 95%: 57.0–84.0%) and accuracy (89.16%, CI 95% 82.19–94.1%) were observed in the case of the Vazyme SARS-CoV-2 Antigen Detection Kit (Fig 2). The poorest performance was observed in the case of the VivaDiag SARS-CoV-2 Ag Rapid Test (sensitivity 43.0%, CI 95%: 26.0–61.0%). Specificity was 100% when testing with the VivaDiag SARS-CoV-2 Ag Rapid Test (CI 95%: 96.0–100%), Vazyme SARS-CoV-2 Antigen Detection Kit (CI 95%: 95.0–100%), Roche SARS-CoV-2 Rapid Antigen Test (CI 95%: 97.0–100%), Helix i-SARS-CoV-2 Ag Rapid Test (CI 95%: 93.0–100%), Healgen Coronavirus Antigen Rapid Test (CI 95%: 93.0–100%) and Abbot COVID-19 Ag Rapid Test (CI 95%: 98.0–100%). The lowest specificity (79.0%, CI 95%: 66.0–88.0%) and accuracy (70.1%, CI 95%: 59.96–78.98%) were calculated for Humasis COVID-19 Ag Test.

The overall positive predictive value (PPV) for all Ag-RTDs was 94.0% (CI 95%: 90.0–96.0%), while it was between 61.0% (Humasis COVID-19 Ag Test) and 100% (Roche SARS-CoV-2 Rapid Antigen Test, Helix i-SARS-CoV-2 Ag Rapid Test, Healgen Coronavirus Antigen Rapid Test, VivaDiag SARS-CoV-2 Ag Rapid Test and Vazyme SARS-CoV-2 Antigen Detection Kit) for the individual tests. The overall negative predictive value (NPV) is 79.0% (CI 95%: 77.0–82.0%) and between 67.0% (CI 95%: 55.0–77.0%, CLINITEST Rapid COVID-19 Antigen Test) and 85.0% (CI 95%: 76.0–92.0%, Vazyme SARS-CoV-2 Antigen Detection Kit) for the individual Ag-RTDs.

**Fig 3:**
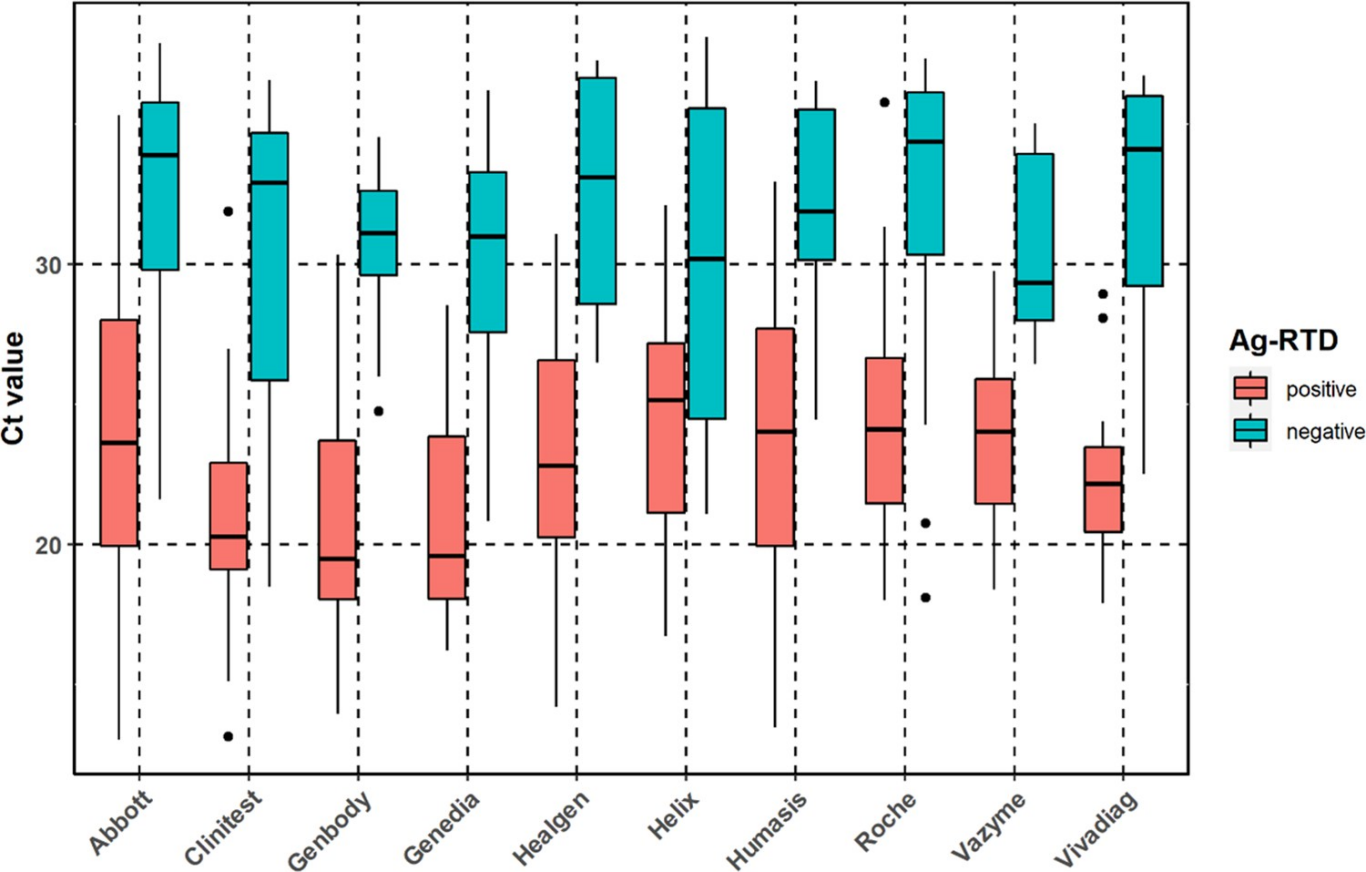
Ct values among true positive (both Ag-RTD and RT-qPCR positive) and false Ag-RTD negative (Ag-RTD negative but RT-qPCR positive) samples by Ag-RTDs.

We also compared the Ct values among true positive (both Ag-RTD and RT-qPCR positive) and false Ag-RTD negative (Ag-RTD negative but RT-qPCR positive) samples by Tukey’s test adjusted for multiple comparison (Fig 3). We found the differences in mean significant in the case of all rapid tests (*p < 0*.*0001*). Overall mean Ct values were 23.4 (N = 318, SD 4.55, CV% 19.5%) and 31.7 (N = 251, SD 4.47, CV% 14.1%), median Ct values were 22.9 (IQR 6.80) and 32.9 (IQR 6.73), minimum and maximum Ct values were 13.0 and 35.8, as well as 18.1 and 38.1 in the case of true positive and false negative samples, respectively. Data are shown in Table 3A–3C.

**Table 3:** Mean, median, minimum, and maximum Ct values with 95% CI observed in the case of each Ag-RTD.

**A**								
	**Abbott**		**Clinitest**		**Genbody**		**Genedia**	
	**positive (N = 90)**	**negative (N = 86)**	**positive (N = 23)**	**negative (N = 25)**	**positive (N = 25)**	**negative (N = 15)**	**positive (N = 21)**	**negative (N = 17)**
**Ct**								
Mean (SD, CV%)	24.3 (5.26, 21.7%)	32.6 (4.10, 12.6%)	21.1 (4.14, 19.6%)	30.3 (5.29, 17.4%)	20.7 (3.71, 17.9%)	30.6 (3.04, 9.9%)	21.1 (3.58, 17.0%)	30.3 (4.41, 14.6%)
Median (IQR)	23.6 (8.08)	33.9 (5.98)	20.3 (3.82)	32.9 (8.83)	19.5 (5.68)	31.1 (3.03)	19.6 (5.79)	31.0 (5.73)
Min, Max	13.0, 35.3	21.6, 37.9	13.2, 31.9	18.5, 36.6	14.0, 30.4	24.8, 34.5	16.2, 28.6	20.8, 36.2
**B**								
	**Healgen**		**Helix**		**Humasis**		**Roche**	
	**positive (N = 19)**	**negative (N = 14)**	**positive (N = 24)**	**negative (N = 20)**	**positive (N = 20)**	**negative (N = 16)**	**positive (N = 47)**	**negative (N = 26)**
**Ct**								
Mean (SD, CV%)	23.4 (4.93, 21.1%)	32.5 (4.15, 12.8%)	24.5 (3.94, 16.1%)	30.4 (5.51, 18.1%)	24.1 (5.53, 23.0%)	32.0 (3.80, 11.9%)	24.4 (4.01, 16.5%)	32.3 (5.15, 15.9%)
Median (IQR)	22.8 (6.32)	33.1 (8.08)	25.2 (6.04)	30.2 (11.1)	24.0 (7.76)	31.9 (5.37)	24.1 (5.20)	34.4 (5.80)
Min, Max	14.2, 31.1	26.5, 37.3	16.7, 32.1	21.1, 38.1	13.5, 33.0	24.5, 36.6	18.0, 35.8	18.1, 37.4
**C**								
	**Vazyme**		**Vivadiag**		**Overall**			
	**positive (N = 34)**	**negative (N = 13)**	**positive (N = 15)**	**negative (N = 19)**	**positive (N = 318)**	**negative (N = 251)**		
**Ct**								
Mean (SD, CV%)	23.8 (2.97, 12.5%)	30.5 (3.29, 10.8%)	22.3 (3.20, 14.3%)	32.0 (4.78, 14.9%)	23.4 (4.55, 19.5%)	31.7 (4.47, 14.1%)		
Median (IQR)	24.0 (4.45)	29.3 (5.93)	22.2 (3.03)	33.5 (6.74)	22.9 (6.80)	32.9 (6.73)		
Min, Max	18.4, 29.8	26.4, 35.0	17.9, 28.9	22.5, 36.8	13.0, 35.8	18.1, 38.1		

**Fig 4:**
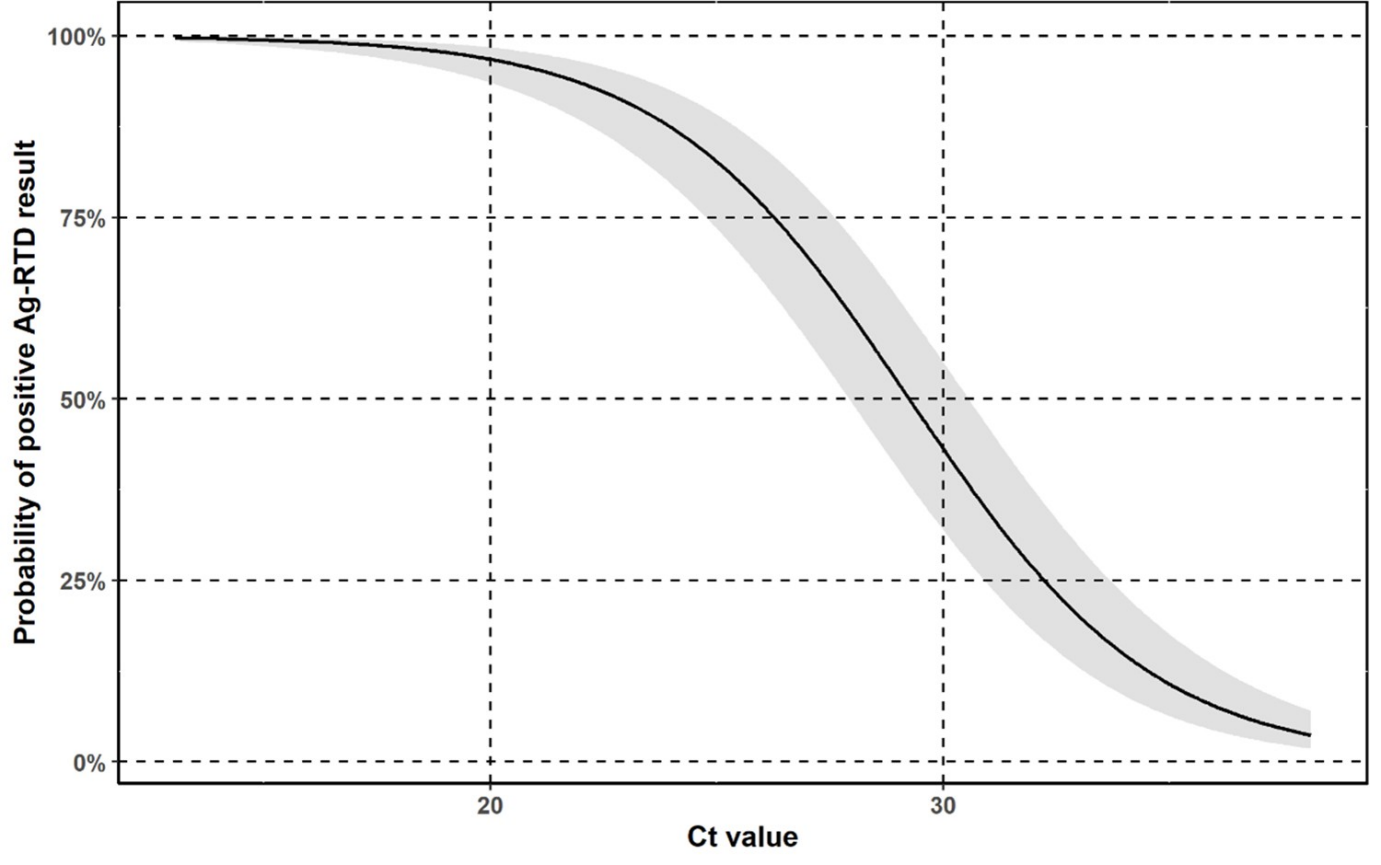
Probability of a positive Ag-RTD result by positive RT-qPCR Ct values. Probability of a true positive Ag-RTD test among Ct values with its 95% confidence interval.

The probability of true positive Ag-RTD test decreased with increasing Ct value with an odds ratio of 0.69 (95% confidence interval: 0.65–0.73 pro 1 Ct value) versus false negative Ag-RTD test. The probability of true positive Ag-RTD test was estimated based on a logistic regression model as shown in Fig 4.

**Fig 5:**
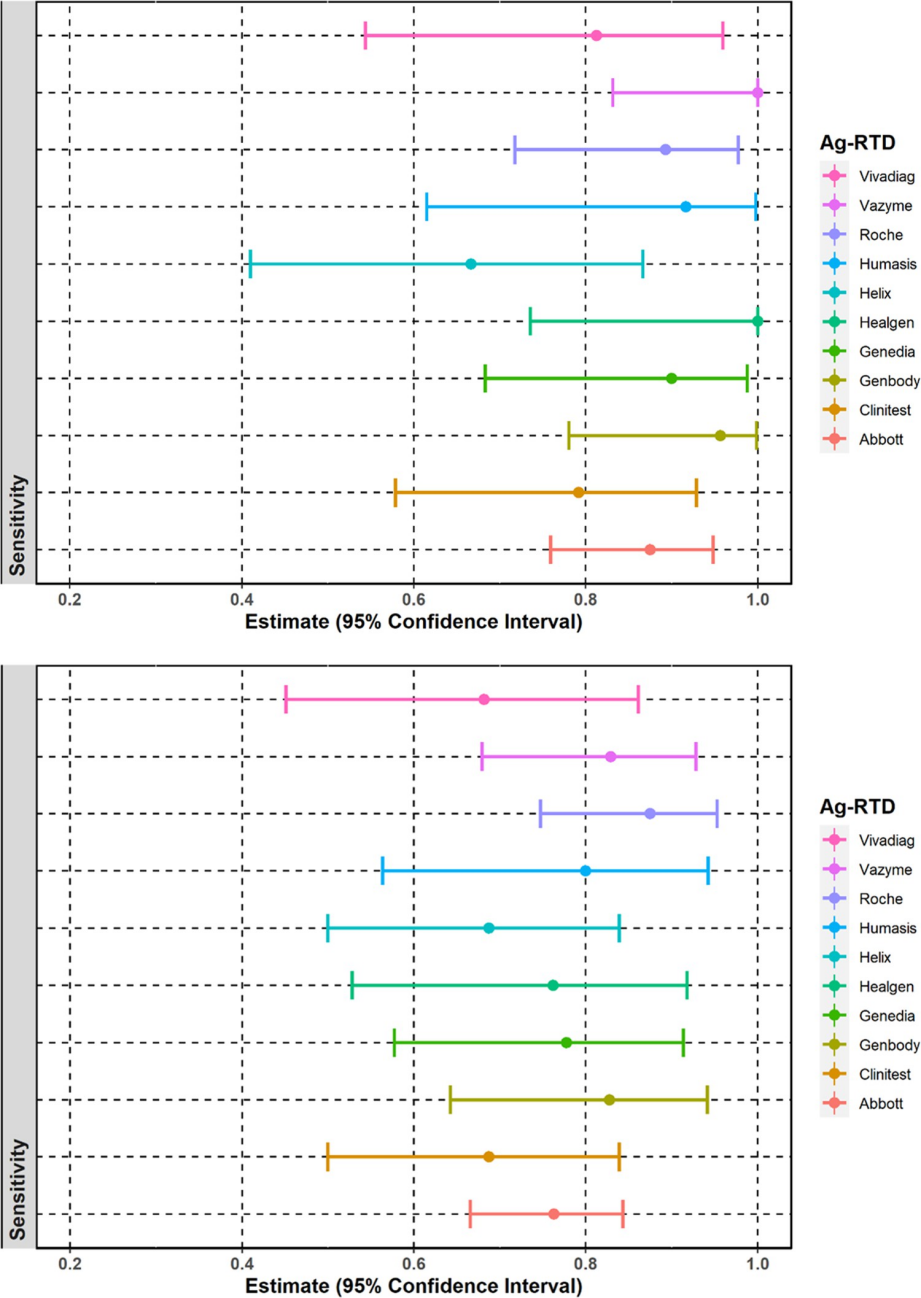
Sensitivity of the ten different Ag-RTDs **A.** strongly positive samples (Ct ≤ 25), **B.** positive samples with Ct ≤ 30.

Based on this probability the sensitivity of each Ag-RTD was also calculated in the case of RT-qPCR positive samples with Ct values ≤ 25 and ≤ 30 with 95% confidence interval. Results are shown in Fig 5A and 5B.

Overall sensitivity of the Ag-RTDs in the case of strongly positive samples (Ct ≤ 25) was 88.1% (CI 95%: 66.5–96.9%) based on 229 tested samples. Highest sensitivity (100%) was observed in the case of the Healgen Rapid COVID-19 Antigen Test (n = 12, CI 95%: 73.5–100.0%) and Vazyme SARS-CoV-2 Antigen Detection Kit (n = 20, CI 95%: 83.2–100.0%) (Fig 5) with 100% overall specificity. Sensitivity of the GenBody COVID-19 Ag Test in the case of strongly positive samples was also above 95% (n = 23, sensitivity 95.7%, CI 78.1–99.9%). The poorest performance was observed in the case of the Helix i-SARS-CoV-2 Ag Rapid Test (n = 18, sensitivity 66.7%, CI 95%: 41.0–86.7%). In the case of samples with Ct value ≤ 30 overall sensitivity was 76.9% based on 369 RT-qPCR positive samples (CI 95%: 58.6–89.8%). The best performance was observed with the Roche SARS-CoV-2 Antigen Rapid Test (n = 48, sensitivity 87.5%, CI 95%: 74.8–95.3%). Other Ag-RTDs with sensitivity over 80% in case of Ct ≤ 30 are the GenBody COVID-19 Ag Test (n = 29, sensitivity 82.5%, CI 95%: 64.2–94.2%), the Vazyme SARS-CoV-2 Antigen Detection Kit (n = 41, sensitivity 82.9%, 95% CI: 69.7–92.8%) and the Humasis COVID-19 Ag Test (n = 20, sensitivity 80%, CI 95%: 56.3–943%);, however, overall specificity of the Humasis test was only 79% (CI 95%: 66–88%).

## Discussion

In most regions globally a decline in reported positive and fatal cases is observed mainly due to vaccination, but some countries such as India, Colombia, Malaysia and Indonesia are still facing a severe third wave of the SARS-CoV-2 pandemic. The emergence of new, highly virulent, and potentially vaccine escape SARS-CoV-2 variants indicates an acute risk for the fourth wave as previously experienced. The B.1.351 South African variant was first reported in Hungary in February and the P.1 Brazil variant was imported at the beginning of June 2021 [9–12]. The emergence of the most recent B1.617 Indian variant has been reported by several countries, in Hungary the first two cases occurred at the end of May [3, 13]. With potentially rapidly increasing number in positive and fatal cases, early diagnosis of infected individuals is essential to control the rapid spread of the virus. Although RT-qPCR test is the recommended and most reliable diagnostic tool, antigen-based immunochromatographic rapid tests can provide a much faster and cost-effective method for on-the-spot diagnosis of COVID-19. During the last year over 160 different commercial tests became available from different manufacturers [14]. However, there are concerns about the overall sensitivity rates due to possible high number of false negative results associated with lower viral loads. Performance of several SARS-CoV-2 rapid antigen tests has been evaluated by many countries, leading to similar conclusions. On 21 January 2021, the European Commission Member States unanimously agreed on a Council recommendation setting a framework for the use of Ag-RTDs and the mutual recognition of COVID-19 test results across the EU [15]. The Council recommendation called on Member States to also agree on a common list of COVID-19 rapid antigen tests, whicht are considered appropriate for use and are in line with countries’ testing strategies, such as carrying a CE marking, meet the minimum performance requirements of ≥ 90% sensitivity and ≥ 97% specificity and have been validated by at least one Member State. Of the 10 evaluated Ag-RTDs in this study, 9 tests are on the European Commission’s common list of COVID-19 rapid antigen tests, while the Helix i-SARS-CoV-2 Ag Rapid Test is not recognized. Although, several studies engaged in evaluating antigen based RTD performance, none of them has tested as many types of devices with a relatively large number of samples using as our laboratory.

Of the Ag-RTDs that were not available to us, Mak et al. determined the performance of the BIOCREDIT COVID-19 rapid test using a ten-fold serial dilution of a SARS-CoV-2 virus isolate and respiratory samples from confirmed COVID-19 patients taken directly into viral transport media (VTM) or phosphate-buffered saline (PBS). Based on the result of 160 respiratory samples, the rapid test detected between 28.6–81.8% of the RT-qPCR positive, very high viral load (Ct < 18.49) samples, but only 0–21.1% of the samples with Ct values between 18.69 and 31.12. Limit of detection was Ct = 28.47 in the case of the virus isolate [16]. The COVID-19 Ag Respi-Strip (Coris Biocencept, Gembloux, Belgium) device was evaluated by Scohy et al. using 148 nasopharyngeal samples of hospitalized patients and compared to the RT-qPCR method. The overall performance was similar to the BIOCREDIT rapid test with a sensitivity of 30.2% (95% CI: 21.7–39.3%) and a specificity of 100% [17]. Laboratory evaluation of the STANDARD Q COVID-19 Ag Test (SD Biosensor, Gyeonggi-do, Republic of Korea) was performed by Nalumansi et al. using 262 nasopharyngeal swab samples from suspected COVID-19 patients. Based on their results sensitivity and specificity were 70.0% (95% CI: 60–79%) and 92% (95% CI: 87–96%), respectively; accuracy was 84% (95% CI: 79–88%). In the case of the samples with RT-qPCR Ct values ≤ 29, sensitivity increased up to 92% [18]. In our study, we evaluated the GenBody COVID-19 Antigen Test and the Roche SARS-CoV-2 Rapid Antigen Test as well, which are from two different distributors but the same manufacturer as the STANDARD Q COVID-19 Ag Test (SD Biosensor, Gyeonggi-do, Republic of Korea) and found very similar results as Nalumansi et al. Based on the result of 98 samples, the overall sensitivity and specificity of the GenBody Ag-RTD test were found 62% (95% CI: 46–77%) and 90% (95% CI: 79–96%), respectively with accuracy of 78.57% (95% CI: 79–96%). In the case of samples with Ct < 30, sensitivity increased up to 81%. In the case of the Roche Ag-RTD test the overall sensitivity and specificity was 64% (95% CI: 52–75%) and 100% (95% CI: 97–100%) while accuracy was 86.94% (95% CI: 81–91%) based on 199 swab samples. In the case of samples with Ct ≤ 30, sensitivity increased up to 82.5%. Diagnostic performance of Abbott’s Panbio COVID-19 Ag Rapid Test (Abbott Rapid Diagnostics) was also evaluated by Landaas et al. in Norway. Their sampling strategy was identical to ours: one nasopharyngeal swab sample taken for RT-qPCR and another for rapid testing at the testing station. A total of 3,991 samples were examined in their study, collected from a single COVID-19 testing station in Oslo and municipality physicians from Norwegian regions with recent COVID-outbreaks. According to their results, the overall sensitivity and specificity of the test were 74.4% (95% CI: 49–79%) and 99.9% (95% CI: 99.7–99.9%), respectively. Among symptomatic patients, sensitivity increased up to 78.9% (95% CI: 73–84%), while in asymptomatic cases it decreased to 55.3% (95% CI: 41–69%). An increase in sensitivity was also observed among samples with Ct ≤ 30 (83.8%; 95% CI: 78–88%) [20]. In our study (based on a total sample number of 543 samples) the overall sensitivity and specificity of the Abbott’s Panbio COVID-19 Ag Rapid Test was 51% (95% CI: 44–59%) and 100% (95% CI: 98–100%), respectively. In case of samples with Ct ≤ 30 sensitivity increased up to 76% (95% CI: 67–84%). Torres et al. determined the performance of the CLINITEST Rapid COVID-19 Antigen Test (Siemens Healthineers, Erlangen, Germany) based on 270 samples collected from patients with suspected COVID-19 infection and their contacts, using a sampling strategy similar to ours; one nasal swab obtained for RT-qPCR and another for rapid testing [21]. According to their data, in the case of symptomatic patients the sensitivity and specificity were 80.2% (95% CI: 70.9–87.1%) and 100% (95% CI: 95.8–100%), respectively, while in asymptomatic patients, sensitivity decreased to 60.0% (95% CI: 40.7–76.6%). When evaluating only strong positive samples (Ct ≤ 25), sensitivity increased up to 95.5% (95% CI: 89–98.2%). In our study the CLINITEST Rapid COVID-19 Antigen Test was also evaluated, however, we obtained different results from Torres et al. The overall sensitivity and specificity were 48% (95% CI: 33–63%) and 98% (95% CI: 90–100%), respectively. Sensitivity in the case of samples below Ct value 25 was 79% (95% CI: 58–93%). When comparing sensitivity, we can see that 95% CI ranges overlap in the case of most evaluated Ag-RTDs (overall sensitivity 56% (95% CI: 52–60%), indicating no significant difference (Fig 2). Regarding specificity, 95% CI ranges overlap in the case of the Humasis Ag-RTD and the GenBody Ag-RTD, but not with the other eight Ag-RTDs indicating significant difference; however, the results are not corrected for multiple comparisons. We also calculated positive and negative predictive values for each rapid test, however, comparing them may be misleading as PPV and NPV are dependent from the prevalence that is different in case of the Ag-RTDs.

It is important to note that our study has no bias of freezing and thawing, as two separate samples were obtained on field from different parts of the nostrils and pharynx of every enrolled patient. The first was used for molecular testing and was taken immediately into the NPHC laboratory to perform RT-qPCR, and another sample for rapid testing was carried out on the spot by trained health care professionals. However, as errors can occur during the sampling procedures, sample amounts taken from one individual for the two different tests may vary. Another limitation of the present study is that the Abbott’s Panbio COVID-19 Ag Rapid Test was available in a much higher number (N = 546) compared to the others (N = 87–199). Second, clinical symptoms were available only for half of the enrolled volunteers; thus, there was no statistical relevance in comparing the symptoms with the rapid test results.

In conclusion, it is easy and convenient to use antigen-based RTDs as a tool for point-of-care testing among symptomatic patients; however, on-field applicability for screening of asymptomatic individuals in schools, offices or factories remains concern. In the case of non-clinical screening, by designating the appropriate test, the potentially highest sensitivity matching with highest specificity should be considered. These Ag-RTDs for screening symptomatic individuals are easier to identify, and take less time to interrupt viral transmission routes, especially in the cases of high community risk scenarios. Furthermore, such independent evaluation studies provide help to the stakeholders at the European Health Security Committee, local authorities and institutions to make the best probable decision.

## Supporting information

S1 Dataset (XLSX)
